# Hemp Seed Hull and Cellulose Acetate Thermoplastic Biocomposites and Their Properties

**DOI:** 10.3390/molecules31091453

**Published:** 2026-04-28

**Authors:** Ramune Rutkaite, Joana Bendoraitiene, Giedruna Pavuolyte, Laura Peciulyte, Dovile Liudvinaviciute, Paulius Barvainis, Visvaldas Varzinskas

**Affiliations:** 1 Department of Polymer Chemistry and Technology, Kaunas University of Technology, Radvilenu Rd. 19, LT-50254 Kaunas, Lithuania; joana.bendoraitiene@ktu.lt (J.B.); giedruna.pavuolyte2@gmail.com (G.P.); laura.peciulyte@ktu.lt (L.P.); dovile.liudvinaviciute@ktu.lt (D.L.); barvainispaulius@gmail.com (P.B.); 2 Centre of Advanced Packaging Materials and Technology Research, Kaunas University of Technology, LT-51424 Kaunas, Lithuania; visvaldas.varzinskas@ktu.lt

**Keywords:** hemp seed hulls, chemical modification, cellulose acetate, thermal extrusion, biocomposites

## Abstract

The study explores the potential of thermoplastic biocomposites made from cellulose acetate, modified hemp seed hull particulate fillers and environmentally friendly plasticizer triacetin. Emphasizing the environmental advantages of utilizing natural materials, the research demonstrates the impact of different hemp hull chemical modification, such as alkali treatment or acetylation, on the mechanical properties of the resultant composites. Hemp seed hulls treated with 4–16% NaOH solution were studied using SEM imaging, FT-IR, XRD and chemical composition analyses. The study showed that alkaline treatment of hemp seed hull particles improved the mechanical properties of biocomposites, and the optimum concentration of NaOH solution ranged from 8 to 12%. The tensile modulus increased by 17–24%, and the tensile strength improved by 21–23% in biocomposites containing HH8 and HH12 fillers, while hardness increased by approximately 11–13%. It was also demonstrated that alkaline treatment of hemp seed hull fillers accelerated the biodegradation of biocomposite extrudates under aerobic conditions in a standard aqueous medium. Overall, the results demonstrated the potential of alkaline-treated hemp seed hulls as fillers in composite bioplastics.

## 1. Introduction

Most of conventional plastics are derived from fossil fuels, and if not properly managed at the end of their life cycle, they can accumulate in the environment, contributing to increased greenhouse gas emissions and pollution. As a result, alternative materials such as bio-based, biodegradable, and compostable plastics are being explored as more sustainable options to replace fossil-based, non-biodegradable polymers. In this context, biologically sourced materials suitable for the production of thermoplastic biocomposites are of particular interest. Cellulose, the most abundant polysaccharide in nature, represents a highly valuable renewable resource, and its utilization in the development of sustainable materials offers significant potential. Integrating cellulose-based components, such as modified cellulose and agricultural by-products like hemp seed hulls, into biocomposite formulations not only reduces reliance on petroleum-derived polymers but also contributes to circular-economy strategies by valorizing biomass residues.

Cellulose acetate (CA) is among the most widely used cellulose derivatives. Pure CA is considered a valuable biopolymer due to its optical clarity and toughness. However, its practical application is limited by the narrow gap between its melting point and thermal degradation temperature. CA has a relatively high melting temperature, typically above 200 °C, due to strong intermolecular hydrogen bonding, and also exhibits a low melt-flow index (2.1 g/10 min at 230 °C, 2.16 kg) [1]. As a result, thermal decomposition can begin shortly after melting, making melt processing challenging. To overcome these issues, plasticizers are commonly added. Plasticizers improve the melt processability of CA and also influence its thermal and mechanical properties [2]. However, plasticization alone cannot solve all of CA’s inherent drawbacks. Even by using plasticizers, cellulose acetate remains relatively hydrophilic, brittle, and lacks sufficient mechanical strength and barrier properties under high-temperature or high-humidity conditions. This highlights the need for further modification of such compositions, e.g., by reinforcing with suitable fillers in order to expand their application potential [3].

Improvement of the chemical and physical properties of biopolymers can be achieved through the formation of composites. Fillers can significantly influence appearance, as well as electrical, thermal, and mechanical properties, including impact resistance, tensile strength, and fatigue resistance [4]. Lignocellulosic biomass fillers are the most widely used reinforcements in biocomposite production and typically originate from fibres such as flax, hemp, ramie, kenaf, jute, pineapple leaf fibres, abaca, sisal, palm, and agave. Natural fibres can be extracted from plant stems (bast fibres), leaves, seeds, fruits, wood, or cereal straw [5]. In contrast, plant shives and hulls remain less explored and underutilized, despite their abundance as agricultural byproducts and their potential for incorporation into value-added biocomposite materials. Hemp and its industrial products are considered one of the most promising resources for developing sustainable food and non-food materials [6]. From an agricultural perspective, hemp cultivation offers several advantages: it has a short cropping cycle [7] and requires significantly fewer pesticides and less water compared with cotton, one of the most common fibre-producing plants. Moreover, all parts of the hemp plant, including its leaves, stalks, roots, and seeds, can be utilized, leaving no waste [8].

Researchers have reported a range of chemical pre-treatment methods aimed at strengthening the interfacial adhesion between fibre surfaces and the polymer composite matrix. The reagents commonly employed for the chemical modification of hemp fibres typically contain functional groups capable of reacting with the free hydroxyl groups on the fibre surface. This reaction reduces fibre hydrophilicity and enhances compatibility with polymer matrices [9,10]. Numerous chemical treatments have been reported in the literature, such as alkaline or silane treatment, maleic anhydride coupling, acetylation, and graft polymerization, recognized as the most widely used and effective fibre-modification techniques [9,11,12,13].

Acetylation is applied to natural fibres to decrease their hygroscopic nature, thereby improving dimensional stability and increasing resistance to environmental degradation.

It is a widely used esterification approach in which acetic anhydride reacts with the fibre surface to replace hydroxyl groups with acetyl groups [14]. This modification improves fibre–matrix adhesion by enhancing interfacial bonding, which in turn results in better mechanical properties of the resulting composites, including improvements in tensile, flexural, and compressive strength, improvement of both dimensional stability and water resistance of the polymeric materials [9,12].

Meanwhile, a review of the papers concerning the alkaline treatment of natural fibre fillers indicates a broad range of sodium hydroxide concentrations, typically ranging between 1 and 20 wt%. Notably, optimal fibre performance is frequently reported at NaOH concentrations near 5 wt%, suggesting the existence of a potential “sweet spot” that balances structural modification with preservation of fibre integrity [14]. In most studies, hemp fibres are immersed in aqueous NaOH solution for a defined time, allowing alkali to react with hydroxyl groups in the amorphous regions of the fibres, disrupting hydrogen bonding networks, partially depolymerising cellulose chains, and exposing shorter crystallites [9,15,16]. This process decreases the hydrophilic character of the fibres and improves their compatibility with polymer matrices. Furthermore, alkaline treatment facilitates the partial elimination of non-cellulosic constituents such as hemicellulose, lignin, and pectins, as well as surface impurities including waxes and oils, thereby increasing fibre surface roughness [9]. This increase in effective surface area, combined with the formation of a roughened fibre texture, provides more sites for mechanical interlocking with the polymer matrix, ultimately enhancing interfacial adhesion. Partial removal of hemicellulose also leads to an enhanced molecular orientation, promoting tighter packing of cellulose chains within the fibre structure and increased crystallinity [9]. Numerous studies demonstrate that alkaline treatment improves the mechanical properties of hemp fibres; however, optimization of parameters such as NaOH concentration, treatment time, and temperature is crucial to achieve maximum effectiveness. For instance, variations in NaOH concentration from 0.8% to 8% have shown strong effects on mechanical performance: the highest tensile strength (1064 MPa) was achieved using 6 wt% NaOH solution, while the maximum Young’s modulus of 65 GPa was observed at 4 wt% NaOH solution conditions, compared with 591 MPa and 38 GPa for untreated fibres, respectively [9]. These findings suggest that the mechanical properties of alkali-treated hemp fibres are strongly governed by NaOH content, as it dictates the extent of fibre modification and structural reorganization.

Modification of hemp fibres with 5% and 18% NaOH solutions at ambient and boiling temperatures for varying treatment durations has been shown to produce fine fibres with great flexibility and reduced water retention capacity, attributable to chemical composition changes [9]. Although tensile properties improved under some treatment conditions, a drastic decline in mechanical performance was observed for fibres treated with 18% NaOH, indicating that excessively high alkali concentrations can cause severe fibre degradation.

Studies of natural waste-based fibres such as rice straw, rice husk, corn husk, hemp residues, and buckwheat husks show that alkali treatment can improve fibre–matrix adhesion by removing lignin, hemicellulose and surface impurities, increasing fibre roughness and enhancing mechanical interlocking [17,18,19,20]. Consequently, composites produced with treated fibres often exhibit higher tensile and flexural strength. Overall, natural waste fibres, whether untreated, mercerized, or otherwise modified, offer promising reinforcement potential for biocomposites, especially when interfacial adhesion is optimized.

Agricultural waste-derived fillers, such as buckwheat hulls, coconut husk fibres, and hemp residues, demonstrate promising potential for incorporation into polymer matrices, enabling the development of biocomposites for various applications [19,21,22]. Moreover, natural fillers provide attractive aesthetic qualities, such as a wood-like appearance and pleasant colour, making these materials appealing for semi-structural or design-focused applications. These materials are especially attractive due to their renewability and competitive performance compared to synthetic materials. For instance, biocomposites produced using buckwheat hull fillers in common polymers such as polyethylene and polylactic acid have shown acceptable mechanical performance [21]. They present strong potential for consumer products, including furniture accessories, decorative elements, and garden equipment. Meanwhile, treated coconut husk fibre reinforced polyester composites have demonstrated excellent acoustic absorption performance, with sound absorption coefficients reaching up to 0.98 at 2.25 kHz frequency [22]. Such properties indicate that coconut husk composites are highly suitable for sound-absorbing elements in buildings.

Hemp residues can be successfully incorporated into polymer matrices [19,23,24]. The study results show that hemp hurd or fibre reinforced polymers can exhibit favourable mechanical, thermal, and acoustic properties, making them a promising sustainable alternative to conventional plastics or wood-based materials.

However, hemp seed hulls remain a widely available yet underexplored agricultural by-product, and, to the best of our knowledge, there is no literature data addressing their application in biocomposites, whether with conventional plastics or bioplastics. The composition of hemp hurd, fibre, and hulls differs mainly in the content of cellulose, hemicellulose, and lignin, and such changes can affect the properties of formed biocomposites. Differences in composition and the lack of literature on the potential use of hulls in biocomposites prompted us to investigate cellulose acetate-based composites incorporating hemp seed hulls as biofillers. Furthermore, the chemical treatment applied to the biofiller is a crucial factor that may affect the properties of the composites, particularly essential characteristics such as mechanical performance and biodegradation behaviour. Therefore, the aim of this study was to chemically modify hemp seed hulls via alkaline treatment and/or acetylation with acetic anhydride, and to investigate the resulting particles as biofillers in thermoplastic cellulose acetate composites, while also evaluating the mechanical performance and biodegradability of the produced biocomposites.

## 2. Results and Discussion

### 2.1. FT-IR Characterization of Raw and Chemically Modified Hemp Seed Hulls

Natural hemp seed hulls (HH) were modified through several sequential operations, including milling and sample fractionation, followed by one or two chemical treatment steps involving alkaline hydrolysis with sodium hydroxide solution and/or acetylation with acetic anhydride. First natural HH was cleaned of impurities, milled into small particles, and fractionated to obtain the particles within a 0.16–0.63 mm size range. The fractions treated with 8% NaOH solution and acetic anhydride were denoted as HH8 and HH/Ac, respectively. Meanwhile, applying alkaline treatment and then acetylation resulted in the formation of the double-modified HH8/Ac sample. Alternatively, a water-washed HH sample (HH0) was produced to examine the effect of water treatment on the composition and functional properties of the modified HH.

**Figure 1:**
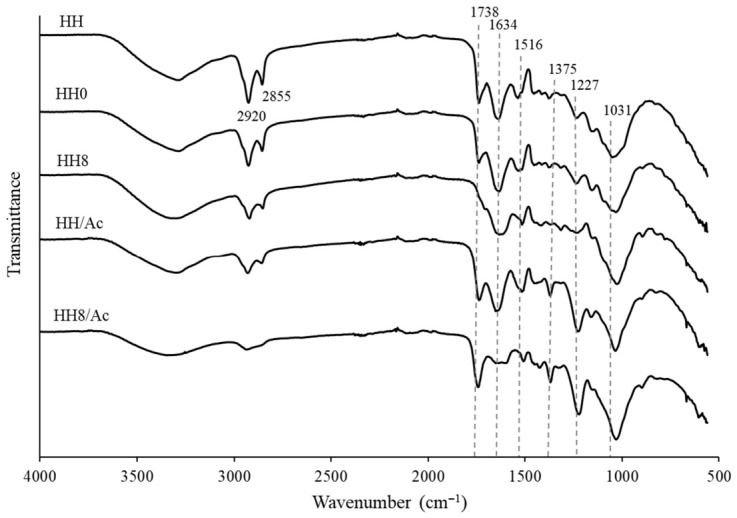
FT-IR spectra of natural HH (HH), water-washed HH (HH0), alkali-treated HH (HH8), acetylated HH (HH/Ac), and alkali-treated and acetylated HH (HH8/Ac) samples.

FT-IR spectroscopy was applied to characterize the prepared samples and evaluate the changes in their structure and functional groups during chemical modification (Figure 1). It is well known that plant biomass is primarily composed of varying amounts of cellulose, hemicellulose, and lignin, along with minor quantities of proteins, pectins, lipids, and ash [25]. The broad absorption band at 3200–3600 cm^−1^ with the peak centred at around 3290 cm^−1^ was present in all spectra (Figure 1). This peak corresponds to O–H stretching vibrations associated with cellulose, and may also include contributions from hydrogen bonds in water molecules adsorbed onto the cellulose surface [26,27]. The –CH stretching bands characteristic of cellulose, hemicellulose, and fat [28] were observed at 2920 cm^−1^ and 2855 cm^−1^ in untreated hemp seed hulls. The band associated with carbonyl groups was observed in the range of 1750–1720 cm^−1^, with a sharp absorption peak at 1738 cm^−1^ corresponding to the C=O stretching vibration of triacylglycerol ester linkages [29]. This peak can also be attributed to carbonyl and uronic acid ester groups present in hemicellulose [26]. Protein-related structural features can be identified by characteristic FTIR absorption bands, particularly observed in the amide I region (1600–1700 cm^−1^) and associated with C=O stretching and N–H bending vibrations, and in the amide II region (1510–1580 cm^−1^), arising from N–H bending and C–N stretching modes [30]. Accordingly, the peaks observed at 1634 cm^−1^ and 1533 cm^−1^ in the spectra of the HH samples can be assigned to protein-associated functional groups. The FTIR spectral region characteristic of lignin typically occurs between 1800 and 800 cm^−1^. Absorption peaks appearing in the ranges 1527–1535 cm^−1^ and 1515–1511 cm^−1^ signify the presence of aromatic C=C skeletal vibrations arising from the lignin aromatic ring structure [29,31]. Meanwhile, the absorption band at 1031 cm^−1^ corresponding to C–O–C stretching vibration is associated with lignin polysaccharides.

No difference was distinguished between the spectra of HH and HH0, indicating a similar composition of the samples. However, following the treatment of HH with 8% NaOH solution, the significant reduction in the intensity of the –CH stretching peaks at 2920 cm^−1^ and 2855 cm^−1^ was determined (Figure 1, sample HH8), indicating the dissolution and removal of some hemicellulose and fat components. It was reported that alkali treatment can lead to some delignification and subsequent weakening of hydrogen bonding between cellulose and hemicellulose, affecting hemicellulose dissolution [26]. The fat reduction was further confirmed by a significant decrease in the 1738 cm^−1^ peak intensity (Figure 1, sample HH8). Further, the reduction in the amide band at 1516 cm^−1^ in the spectra of HH8 was observed, suggesting the removal of water-soluble proteins. Therefore, it can be concluded that during alkali treatment with 8% NaOH solution, the HH composition was substantially affected.

The structure of HH samples was further affected during chemical modification with acetic anhydride and the introduction of acetyl groups. Characteristic absorption bands of acetyl groups include the C=O stretching vibration at approximately 1738 cm^−1^, the C–O stretching of ester functionalities in the acetyl group appearing at 1031–1036 cm^−1^, and the C–H bending vibration at 1375 cm^−1^ [27]. As can be seen in Figure 1, the spectra of both acetylated HH (HH/Ac) and HH subjected to alkaline treatment followed by acetylation (HH8/Ac) exhibited an intensified absorption peak at 1738 cm^−1^ and the appearance of a new peak at 1375 cm^−1^, corresponding to the C=O stretching vibration and C–H bending vibration, respectively. In addition, a strongly enhanced aryl ether band at 1227 cm^−1^ indicates that acetylation also occurred at hydroxyl group sites associated with the lignin structure [27]. The latter observation is in agreement with literature data, which confirms that hydroxyl groups of amorphous components such as hemicellulose and lignin readily undergo esterification; meanwhile, the hydroxyl groups in crystalline cellulose are tightly bound through hydrogen bonding, which restricts reagent accessibility and leads to less effective functionalization [9,14].

### 2.2. Testing of Chemically Modified Hemp Seed Hulls as Fillers in Thermoplastic Cellulose Acetate Biocomposites

Several studies highlighted that the alkali treatment of plant biomass leads to the partial removal of lignin, hemicellulose, and pectin, along with surface impurities including waxes and oils that coat the fibre cell wall [15,16]. As a result, fibres exhibit increased surface roughness, which can improve interfacial adhesion in composite materials. It is well established [16] that various surface-modification techniques that enhance fibre–matrix adhesion can also lead to improvements in the mechanical properties of biocomposites. Therefore, chemically modified HH samples were trialled as particulate biofillers in plasticized CA composites. Furthermore, triacetin (TA), an environmentally benign and non-toxic plasticizer, was chosen for the development of thermoplastic CA formulations.

In this work, thermal twin-screw extrusion was adopted as the primary processing approach to produce composites based on thermoplastic cellulose acetate/triacetin and various HH biofillers (HH, HH0, HH8, HH/Ac, H8/Ac). During twin-screw extrusion, the principal processing parameters were continuously monitored, and the key technological parameter, specific mechanical energy (SME) was determined as shown in Table 1.

SME is widely used as a performance indicator to evaluate the energy efficiency of thermal extrusion operations. It quantifies the mechanical energy required per unit mass to deform, shear, and convey material through the system [32]. From the data shown in Table 1, it can be seen that the SME value of the CA/TA formulation is 542.6 ± 22.6 kJ/kg, and is significantly higher compared to those with the filler. The addition of HH fillers resulted in a substantial decrease in SME values, lowering them by a factor of 3–5. High SME values indicate great energy demand associated with material resistance, while low values suggest more efficient processing. Similar tendency for decreased SME values of composites as compared to neat polymer was also observed in PLA composites containing sugar beet pulp [33]. It was shown that the decrease in SME was highly dependent on the filler weight fraction in the composite and was associated with the moisture content in the pulp, which led to the decreased viscosity of the melt and thus lowered SME values. However, in the present study, moisture content was quite low, and significantly lower values were observed for all HH-containing samples. It can be suggested that treatment of HH affected the surface roughness of the filler particles, indicating improved penetration of the plasticizer into the particle surface. Consequently, a lubricating interphase forms around the filler particles, reducing interfacial friction and, as a result, lowering the specific mechanical energy during extrusion. Although the lowest SME values within the range of 110.1–127.4 kJ/kg were determined for cellulose acetate formulations containing chemically modified HH fillers, no significant variation in SME among those samples was observed.

The melt flow rate (MFR) quantifies the flowability of plastics under processing conditions and determines whether the material is suitable for melt processing. Thus, MFR measurements of the biocomposite extrudates with various biofillers were conducted (Table 1) to establish the most promising biofiller to be further investigated. The melting of all biocomposite granules was slightly more difficult compared to the neat matrix without filler (CA/TA), as indicated in the lower MFR values established for biocomposites. It can be observed that chemical modification of HH further reduced the MFR, with acetylation having the strongest effect. In this case, the presence of acetyl groups in both the matrix and the filler structures probably has a decisive influence. Due to the interactions between the acetylated components, the flow of the melt is more difficult, resulting in higher MFR values for CA/TA:HH8/Ac and CA/TA:HH/Ac samples. In contrast, the use of alkaline-treated HH particles in CA composites did not cause such a significant decrease in MFR value, and biocomposites prepared with HH8 filler exhibited satisfactory melt-flow properties due to the improved compatibility and reduced filler-filler interaction during processing, which enhances the fluidity of the composite melt.

Tensile testing of dog-bone composite specimens was performed to evaluate the effect of HH fillers on biocomposite mechanical performance. Mechanical properties were evaluated in terms of tensile strength, tensile modulus, and elongation at break (Table 1). It can be noted that by inclusion of raw HH or water-washed HH0 particles into the CA bioplastic, the tensile strength and elongation at break values were significantly reduced. Similarly, plasticized CA and microcrystalline cellulose composites exhibited reduced tensile strength and elongation at break compared with the neat, plasticized polymer, which was attributed to uneven particle size and distribution within the matrix [34]. In the present study, incorporation of chemically modified HH fillers into the CA/TA matrix led to a modest reduction in tensile strength and a significant increase in tensile modulus. For instance, the increase of 13.3% and 10.8% in tensile modulus was observed for CA/TA:HH8 and CA/TA:HH8/Ac composites, respectively, compared to the blank bioplastic (CA/TA). Similar trends, i.e., increased tensile modulus and strength, flexural modulus and strength, and a great decrease in elongation at break and impact strength, were observed for the composites of CA and bleached (kraft) and non-bleached microcrystalline cellulose and CA composites [35]. Although the flexural properties were not evaluated in our study, it can be presumed that the enhanced stiffness of the developed CA/TA:HH composites would lead to improved structural stability, and hence improved flexural performance, such as flexural modulus. In the present study, in the case of all prepared biocomposites, the elongation at break decreased significantly by 54–64%, although only a slight increase in the hardness was observed. Consistent with previous studies, natural fillers restrict chain mobility, increasing hardness and stiffness while reducing ductility in polymer matrices [36,37]. It can be noted that when evaluating tensile strength, tensile modulus, and hardness parameter values, the CA/TA:HH8 and CA/TA:HH8/Ac biocomposites exhibited the most favourable results. Furthermore, acetylation did not have a significant effect on the mechanical properties of the composites. Therefore, given that the single-stage chemical modification process is more straightforward, the alkali-treated hemp seed hull samples were selected for further investigation of the CA biocomposites, aiming to equilibrate the level of alkali modification of HH and its effect on the mechanical properties of the biocomposites.

### 2.3. Effect of Alkaline Treatment on Hemp Seed Hull Properties

NaOH solutions with concentrations ranging from 4% to 16% were employed to modify the surface of natural hemp seed hulls to improve the interaction between biofiller and polymer matrix, and the mechanical properties of the biocomposites. The impact of alkaline treatment was assessed by analyzing the chemical composition, SEM imaging was used to visualize morphological changes in the particle surface, and XRD analysis was conducted to evaluate the influence of alkali on cellulose crystallinity.

Alkaline treatment is known to induce significant changes in the chemical composition of lignocellulosic biomass, demonstrating the strong affinity of NaOH for amorphous cell wall components [38]. Water-washed HH exhibited low cellulose content (18.7%) and high contents of hemicellulose (24.8%), lignin (34.6%), and protein (12.9%). These findings are consistent with values reported in the literature on HH composition, although some variation is observed due to differences in analytical techniques. Literature values typically report cellulose contents of 22–37%, hemicellulose around 17%, lignin 16–20%, and protein approximately 12% [28,39]. The obtained data show that sodium hydroxide selectively solubilized hemicellulose and partially delignified hemp seed hulls (Table 2) without the loss of cellulose in HH biomass. Therefore, the proportion of cellulose was increased due to the relative enrichment of the solid fraction as other components were removed. For instance, in the HH16 sample, cellulose content increased by more than 110% compared with water-washed HH, as reflected in the extensive removal of hemicellulose (29%), lignin (9%), proteins (55%), and fat (51%) under stronger alkaline treatment conditions. These findings are consistent with the FT-IR spectra data (Figure 1), where reduced intensities of functional groups associated with lignin, hemicellulose, proteins, and fats are evident in the HH8 sample. A minor decrease in ash content can be attributed to the partial leaching of water-soluble mineral components, as well as the loss of fine particulate matter that was dislodged and washed away during alkaline treatment. These findings are consistent with literature data on alkali-treated hemp hurd, where an increase in cellulose content and a decrease in hemicellulose, protein, and ash content were also observed [40].

**Figure 2:**
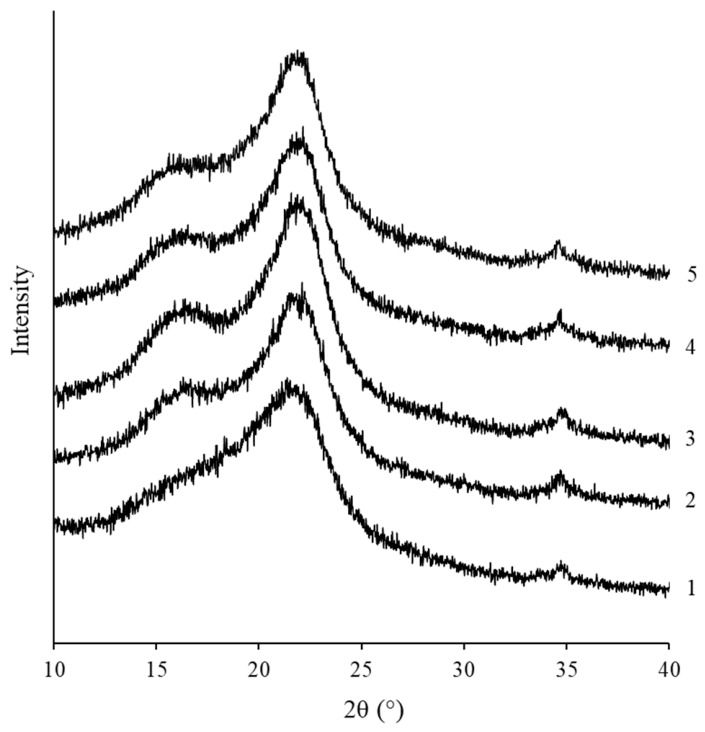
XRD patterns of water-washed HH (1) and alkali-treated HH samples: HH4 (2), HH8 (3), HH12 (4), and HH16 (5).

Since alkaline treatment can induce both compositional and structural reorganization within lignocellulosic biomass, the chemical changes identified in the HH samples were complemented by XRD analysis to determine the extent of crystalline phase modification. The XRD patterns (Figure 2) of alkali-treated HH materials show three distinct broad peaks at 2θ = 22.5°, 16.5°, and 14.5° corresponding to the (200), (110), and ($1\bar{1}0$) crystallographic planes, respectively, and a weaker signal at 34° corresponding to higher-order planes such as (004). These reflections are typical of Cellulose I, the native allomorph of cellulose [41,42].

Literature data [42,43] indicate that treatment with highly concentrated alkali solutions, such as >15–18% NaOH, can disrupt the hydrogen bonding network in Cellulose I, causing fibril rearrangement and resultant cellulose mercerization, during which the native Cellulose I structure is converted into Cellulose II. However, the remaining characteristic peaks in Figure 2 indicate that during the alkali treatment, the Cellulose I was not converted into Cellulose II; instead, the crystallinity of Cellulose I was preserved. It is also important to note that no new diffraction peaks associated with Cellulose II, typically appearing at approximately 2θ ≈ 12° and 20°, were detected.

The increase in crystallinity observed in the XRD curves of alkali-treated HH samples can be attributed to the higher cellulose content in the material. Furthermore, it can be observed that the highest crystallinity was recorded for sample HH8 (Figure 2, curve 3). Alkaline treatment partially removes hemicellulose, leading to increased molecular orientation, improved cellulose chain packing, and enhanced crystallinity [44]. However, at higher NaOH concentrations (>12% NaOH), partial disruption of the crystalline regions may occur, which leads to a decrease in crystallinity again (Figure 2, curves 4, 5).

**Figure 3:**
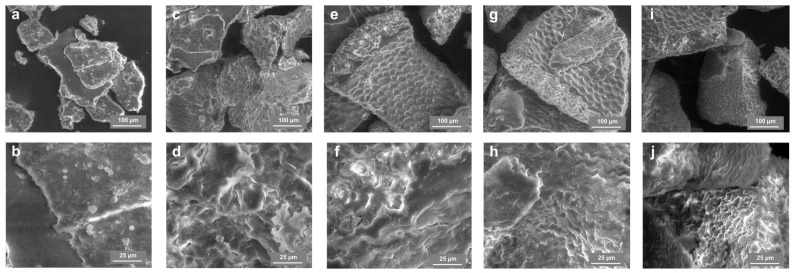
SEM micrographs of HH0 (**a**,**b**), HH4 (**c**,**d**), HH8 (**e**,**f**), HH12 (**g**,**h**) and HH16 (**i**,**j**) samples. SEM images were captured at a magnification of ×500 (**a**,**c**,**e**,**g**,**i**) and ×2000 (**b**,**d**,**f**,**h**,**j**).

The morphological changes in HH particles after the treatment with 4–16% NaOH were assessed by SEM. The surface of water-washed HH was smooth (Figure 3a,b). Meanwhile, significant changes in the HH surface morphology were observed even using low-concentration NaOH solutions (Figure 3c,d). The surface roughness was characteristic of all particles obtained following treatment with a NaOH solution (Figure 3c–j. When using high NaOH concentration and harsh processing conditions, the layers of hemicellulose, proteins, oils, fat, and lignin are removed, causing the partial separation of cellulose microfibrils and increased alkali penetration into the particles [15,16,28]. Therefore, the HH8, HH12 and HH16 particle surfaces exhibited microcracks along with better exposed cellulose fibre bundles (Figure 3e–j). Especially, the effect of using 16% NaOH treatment is obvious when the particles of HH16 were found to be porous like pumice stone with enhanced surface area (Figure 3i,j).

### 2.4. Effect of Alkaline Treatment of Hemp Seed Hulls on Mechanical Properties and Biodegradability of Thermoplastic Biocomposites

An enhancement in the specific surface area of the treated alkali-treated HH particles can promote stronger interfacial adhesion with the biopolymer matrix. Therefore, CA/TA:HH biocomposites were fabricated through thermal twin-screw extrusion using 20% alkali treated HH as biofiller. Mechanical properties, including tensile strength, tensile modulus, elongation at break, and hardness, were evaluated for CA/TA:HH biocomposites incorporating untreated hemp seed hulls and HH chemically modified with NaOH solutions of different concentrations (Table 3).

The optimal mechanical performance was achieved when the hemp seed hulls were treated with the 8–12% NaOH solution, as reflected by the enhanced tensile strength, tensile modulus, and hardness of CA/TA:HH8 and CA/TA:HH12. Similar tendency for increased tensile modulus and strength was observed for alkaline-treated flax fibre-epoxy and sisal fibre-polyethylene composites [45,46]. It was indicated that increased tensile properties were associated with increased roughness of the surface and better adhesion between fibre and matrix. In particular, CA/TA:HH8 extrudate showed an excellent balance of properties, including high tensile strength (35.43 MPa), a significantly increased tensile modulus (1306 MPa), and the highest Shore D hardness value of 74.7, indicating a stiff and mechanically robust sample structure. Meanwhile, CA/TA:HH12 demonstrated the highest tensile strength (36.00 MPa) and the highest tensile modulus (1377 MPa) values, making it the stiffest and strongest material in the series. Thus, biocomposites containing HH8 and HH12 exhibited increases in tensile strength of 21% and 23%, and increases in tensile modulus of 17% and 24%, respectively. The hardness of these biocomposites also improved markedly, by approximately 11–13%. However, stronger alkali treatments may disrupt the fibrillar organization of the biofillers and remove excessive structural components, weakening biofiller–matrix interactions and resulting in inferior biocomposite properties.

As this study intentionally utilized a biodegradable biopolymer matrix, i.e., modified cellulose together with HH biofiller, with the objective of partially substituting traditional polymers with agri-food by-products while maintaining the essential performance characteristics of the biocomposites, the assessment of the biodegradability of such materials is essential. However, biopolymer biodegradation, although expected, depends on numerous factors, including test conditions, the medium used, the diversity and metabolic activity of the microorganisms present, including their sensitivity to the material, and the morphology of the testing samples [47]. The biodegradability of CA/TA:HH0, CA/TA:HH4, and CA/TA:HH16 biocomposite extrudates was evaluated under aerobic conditions in a closed respirometer and was quantified by monitoring oxygen consumption in an aqueous medium inoculated with composted horse manure. The biodegradation of biocomposites was also compared with that of the CA/TA matrix.

**Figure 4:**
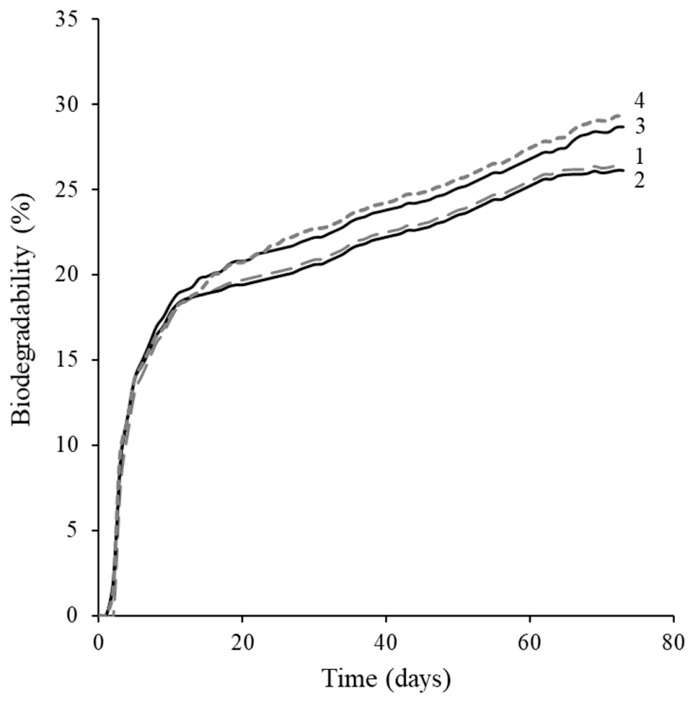
Biodegradation curves for bioplastic extrudates: CA/TA (1), CA/TA:HH0 (2), CA/TA:HH4 (3), and CA/TA:HH16 (4).

As it is shown in Figure 4, the biodegradation of the matrix and the biocomposite containing the water-washed HH filler was very similar (Figure 4, curves 1 and 2), and after 70 days reached approximately 26%. These results differ from those of Buchanan et al. [48], who reported biodegradability exceeding 60% in a liquid medium for cellulose acetate films with a degree of substitution of 2.5 within just eleven days. Furthermore, the biocomposites incorporating alkali-treated HH fillers exhibited comparable biodegradation rates during the first days of testing, but then began to degrade more rapidly. By the end of the experiment, their biodegradation reached approximately 29%. The biodegradation curve exhibited an upward trend in the later stages of testing, suggesting ongoing microbial activity and indicating that the composite materials may continue to degrade beyond the monitored period. This enhanced biodegradation of biocomposites with alkali-treated biofillers is likely related to the removal of hemicellulose, lignin, proteins, fat, and other extractives during the alkaline treatment. It can be concluded that elimination of these components increases surface accessibility and may facilitate microbial attack, thereby accelerating the overall degradation process [49].

## 3. Materials and Methods

### 3.1. Materials

Hemp seed hulls (HH) were donated by Rolandas and Irena Simkai’s subsistence agriculture farm (Kaunas district, Lithuania). Flakes of cellulose acetate (CA), having a degree of substitution of acetate groups of 2.4 and an ash content not exceeding 0.10%, were supplied by Daicel Corporation (Osaka, Japan). Triacetin (TA) (≥99%) was purchased by Sigma-Aldrich Chemie GmbH (Schnelldorf, Germany). Composted horse manure was obtained from UAB Biohumus & Soil (Rokiskis, Lithuania). Unless otherwise stated, all other reagents were of analytical grade and were used as received.

### 3.2. Preparation and Chemical Treatment of Hemp Seed Hulls

Initially, hemp seed hulls (HH) were mechanically milled using a coffee grinder and fractionated by mechanical sieving. The HH were passed through sieves with mesh sizes of 0.16 mm and 0.63 mm, and the particles within the size range >0.16 mm and <0.63 mm were further processed.

The chemical modification of HH was performed by alkaline and/or acetylation treatment (Table 4). Alkaline modification was conducted by mixing thoroughly 100 g of HH with 500 g of 4%, 8%, 12%, or 16% sodium hydroxide solution and maintaining it at room temperature for 24 h. Afterwards, the HH was rinsed with distilled water to reach a neutral pH, filtered, and dried in an oven at 105 ± 1 °C. The prepared alkali-treated samples were denoted as HH4, HH8, HH12 and HH16, accordingly. Meanwhile, sample HH0 was obtained by keeping the original hemp seed hulls in distilled water at room temperature for 24 h and drying afterwards.

Acetylation was carried out by reacting HH with acetic anhydride as the acetylating agent, using sulfuric acid as a catalyst. 10 g of HH were reacted with 50 mL of acetic anhydride and 0.1 mL of concentrated sulfuric acid. After vigorous stirring for several minutes, the mixture was maintained at 30 °C for 24 h. Subsequently, the modified HH were washed with distilled water, filtered, and oven-dried at 105 ± 1 °C. The acetylated HH sample was denoted as HH/Ac, and the double-modified HH was denoted as HH8/Ac.

### 3.3. Analysis of Hemp Seed Hull Samples

#### 3.3.1. Determination of Fat Content

Soxhlet extraction with hexane was employed to determine the fat content of the HH samples. 150 mL of solvent was poured over 5 g of HH placed in a paper thimble and treated in the Soxhlet apparatus for 6 h. The fat content was calculated using the following formula:

(1) $$Fat\left(\%\right)=\frac{\left(m_{f1}-m_{f0})-(m_{f2}-m_{f0}\right)}{m_{f1}-m_{f0}}\cdot 100\%$$

where *m_f_*_0_ is the weight of the empty thimble (g), *m_f_*_1_ is the weight of the thimble with sample before extraction (g), and *m_f_*_2_ is the weight of the thimble with sample after extraction (g).

The dry defatted hemp hulls were kept in a sealed container in a desiccator until further analysis.

#### 3.3.2. Determination of Cellulose and Hemicellulose Content

Prior to the determination of cellulose and hemicellulose amounts in the defatted HH, first, the content of holocellulose was determined. A total of 1.5 g of dry defatted HH sample was weighed and placed into a three-neck round-bottom flask. Then 125 mL of distilled water, 1 mL of glacial acetic acid, and 4 mL of 25% aqueous sodium chlorite solution were added. The flask was equipped with a reflux condenser and placed in a water bath and maintained at 70 °C for 5 h at continuous agitation using a magnetic stirrer. Fresh portions of acetic acid (1 mL) and sodium chlorite solution (4 mL) were added every hour for 4 h. The mixture was then cooled down to room temperature, and the residue was filtered and washed with 500 mL of water until the colour changed from yellow to white, indicating successful delignification. The residue was finally dried at 105 °C ± 1 °C and weighed. The holocelullose content was calculated according to the following equation:

(2) $$Holocellulose\left(\%\right)=\frac{m_{HC0}-m_{HC1}}{m_{HC0}}\cdot 100\%$$

where *m_HC_*_0_ is the initial weight of the dry sample (g), and *m_HC_*_1_ is the weight of the dried sample residue after the delignification (g).

For the determination of cellulose content, 5 g of dry defatted HH sample was weighed and placed into a 250 mL Erlenmeyer flask. The sample was treated with 125 mL of nitric acid ethanol solution (a mixture of 65% *w*/*w* aqueous nitric acid solution and 95% ethanol at a volume ratio 1:4) and boiled under reflux for 1 h. Then the solution was discarded, and a fresh portion of nitric acid ethanol solution was added to continue reflux. The solution was discarded every hour for a total of 4 h, and finally was washed with 1 L of hot distilled water and filtered through a pre-weighed glass filter. The residue was dried in the oven, and the content of cellulose was calculated using the following formula:

(3) $$Cellulose\;\left(\%\right)=\frac{m_{C0}-m_{C1}}{m_{C0}}\cdot 100\%$$

where *m_C_*_0_ is the weight of the sample (g), and *m_C_*_1_ is the weight of the oven-dried sample residue (g).

As holocellulose is the total carbohydrate fraction of cellulose and hemicellulose in the raw material, the amount of hemicellulose was calculated using the following equation:

(4) $$Hemicellulose\;\left(\%\right)=Holocellulose\;(\%)-Cellulose(\%)$$

#### 3.3.3. Determination of Lignin Content

For the determination of lignin content, a two-stage hydrolysis was performed. In the first step, 1 g of dry biomass was treated with 15 mL of 72% sulfuric acid solution at room temperature for 2 h. Subsequently, 200 mL of distilled water was added to dilute the mixture, which was then boiled for another 4 h under reflux. Afterwards, the acid-insoluble lignin fraction was washed with 500 mL of hot distilled water, filtered through a glass filter, and dried in the oven at 105 ± 1 °C. The lignin content was calculated according to the following equation:

(5) $$Lignin\left(\%\right)=\frac{m_{0}-m_{1}}{m_{L0}}\cdot 100\%$$

where *m*_0_ is the weight of the dry glass filter with residue after analysis (g), *m*_1_ is the weight of the empty dry glass filter (g), and *m_L_*_0_ is the initial weight of the sample (g).

#### 3.3.4. Determination of Protein Content

The protein content was quantified using the Kjeldahl method. The protein content is calculated by multiplying the established nitrogen percentage by the conventional conversion factor of 6.25 [50].

#### 3.3.5. Determination of Ash Content

For ash determination, 2 g of dry HH was weighed and transferred to a pre-weighed porcelain crucible, which was further heated on a hotplate for 30 min, by periodically shaking the samples every 5–6 min. After initial carbonization, the crucibles were transferred into a muffle furnace and incinerated at 525 ± 10 °C for 5 h. After ashing, the crucibles were allowed to cool in a desiccator and then weighed. Ash content was calculated using the following equation:

(6) $$Ash\left(\%\right)=\frac{\left(m_{A1}-m_{A0})-(m_{A2}-m_{A0}\right)}{m_{A1}-m_{A0}}\cdot 100\%$$

where *m_A_*_0_ is the weight of the empty crucible (g), *m_A_*_1_ is the weight of the crucible with sample, and *m_A_*_2_ is the weight of the crucible with ash.

### 3.4. Scanning Electron Microscopy (SEM) Analysis

Raw and sodium hydroxide-treated hemp seed hull samples were investigated using a scanning electron microscope FEI Quanta 200 FEG (Brno, Czech Republic). Samples were attached to the metal stubs using adhesive tape and observed at a magnification of 500× and 2000×.

### 3.5. FT-IR Spectroscopic Analysis

Hemp seed hull samples were analyzed using a Frontier (Perkin–Elmer, Waltham, MA, USA) spectrophotometer equipped with a single reflectance horizontal attenuated total reflectance cell with a diamond crystal. FT-IR spectra were recorded in the range from 560 to 4000 cm^−1^ by accumulating 6 scans with a resolution of 4 cm^−1^.

### 3.6. Wide Angle X-Ray Diffraction Analysis

X-ray diffraction (XRD) analysis of the raw and alkali-treated HH samples was carried out using a D8 Advance diffractometer (Bruker AXS, Karlsruhe, Germany) operated at 40 kV and 40 mA. The X-ray beam was filtered using a 0.02 mm Ni filter to select the Cu Kα radiation. Diffraction patterns were collected in Bragg–Brentano geometry employing a Bruker LynxEye fast counting detector. The samples were scanned over a 2θ range of 3–70° with a detector step size of 0.02°.

### 3.7. Preparation of Cellulose Acetate, Plasticizer and Biofiller Formulations

To obtain homogeneous formulations, CA flakes were sieved through a 1.0 mm mesh to remove coarse particles and subsequently dried at 105 °C until a constant mass. First, CA particles (65 wt%) were mixed with a plasticizer TA (35 wt%). Further, the CA/TA composition was mixed with various HH samples (HH0, HH4, HH8, HH12, HH16, HH8/Ac or HH/Ac) to achieve 20 wt% biofiller content in the mixtures. The prepared formulations were stored in sealed containers at room temperature for 24 h prior to extrusion.

### 3.8. Extrusion of Cellulose Acetate, Plasticizer and Biofiller Formulations

The prepared CA, TA and biofiller formulations were processed by extrusion using a co-rotating twin-screw extruder ZE 12 HMI (ThreeTec GmbH, Seon, Switzerland) equipped with six segmented barrels and a length-to-diameter (L/D) ratio of 40:1. Extrusion was carried out at a screw speed of 40 rpm, with material feeding controlled using an ED 12 feeder (ThreeTec GmbH, Seon, Switzerland) set to 3.5% capacity. Extrusion was performed under a temperature profile of 90, 130, 190, 190, 190, and 190 °C applied across the six-barrel segments, progressing from the feed zone to the die. A die containing a single circular orifice with a diameter of 3 mm was employed. The extrudates were subsequently granulated using an SP50 granulator (KraussMaffei Berstorff GmbH, Laatzen, Germany) and stored in hermetically sealed plastic bags prior to subsequent analyses. Specific mechanical energy (SME) was calculated using the following formula [31]:

(7) $$SME=\frac{2\cdot \pi \cdot n\cdot \tau }{60\cdot m}$$

where SME is the specific mechanical energy applied to a system per kg of processed sample (kJ/kg), *n* is the screw speed (rpm), *τ* is motor load or torque (N·m), and *m* is the throughput of the sample (kg/s).

### 3.9. Melt Mass-Flow Rate Determination

The melt mass-flow rate (MFR) of CA/TA and biocomposite extrudates was measured following ISO 1133 (Method A) [51] using a Cflow extrusion plastometer (Zwick/Roell, Ulm, Germany) equipped with a die of 8.000 ± 0.025 mm length and an orifice diameter of 2.095 ± 0.025 mm. The barrel temperature was set to 190 °C with a preheating time of 3 min, and a standard load of 5 kg was applied. Samples were cut at fixed time intervals, with at least five replicates measured per sample. The MFR was reported as the mean value ± standard deviation (SD) and expressed in g/10 min.

### 3.10. Mechanical Testing

Dog bone-shaped specimens (78 mm in length and 2 mm in thickness) were fabricated using a piston injection moulding system (HAAKE MiniJet Pro, Thermo Fisher Scientific, Waltham, MA, USA). The temperature of the injection barrel was maintained at 190 °C, while the mould temperature was set to 20 °C. The biocomposite extrudate granules were loaded into the cylinder and preheated for 3 min. The sample was injected at a pressure of 800 bar for 20 s, followed by a post-pressure step of 200 bar applied for 15 s.

The mechanical behaviour of bone-shaped samples was characterized by tensile testing on a BDO-FBO.5TH machine (Zwick/Roell, Ulm, Germany) according to ASTM D638 standard [52], applying a crosshead rate of 50 mm/min. A minimum of 10 measurements were performed to obtain reliable data, and results are expressed as the mean ± standard deviation (SD).

### 3.11. Hardness Testing

Shore durometer hardness test was conducted according to ASTM D 2240 [53] standard using Shore durometer TI-D, type D (Sauter GmbH, Wutoschingen–Degernau, Germany). Hardness testing of bone-shaped specimens was performed at least at 15 locations on three different samples, and the results were expressed as mean ± standard deviation (SD).

### 3.12. Biodegradation Testing

Biodegradation of CA/TA and biocomposite extrudates in liquid medium was determined under aerobic conditions using a manometric respirometric OxiTop^®^ OC110 controller (WTW GmbH, Weilheim, Germany). The OxiTop control system consisted of a 510 mL sample bottle sealed with a measuring head, a small container containing a CO_2_ absorbent fixed at the bottle neck, and an OxiTop controller for data acquisition. The method relied on automated pressure measurement using a piezoresistive electronic pressure sensor in a sealed bottle under constant temperature conditions. Bottle pressure measurement and biological oxygen demand (BOD, mg/L) calculation were carried out automatically by the system. The standard liquid medium and compost-derived inoculum (3% *v*/*v*) were prepared according to ISO 14851:1999 [54]. The extrudate samples and control sample (microcrystalline cellulose) were added to the prepared test medium and were maintained at 25 ± 2 °C for 70 days. All measurements were conducted in duplicate. Biodegradability was determined by comparing BOD with theoretical oxygen demand (ThOD) and calculated using the following formula:

(8) $$Biodegradation\;(\%)=\frac{{BOD}_{T}-{BOD}_{B}}{ThOD\cdot C_{T}}\times 100$$

where *BOD_T_* (mg/L) is the BOD of the sample in a test bottle, *BOD_B_* (mg/L) is the BOD of the activated sludge (blank sample), *C_T_* (g/L) is the concentration of the sample in test medium, and *ThOD* (mg/g) is the BOD of the sample calculated theoretically by assuming that the polymer completely degraded into CO_2_ and H_2_O.

## 4. Conclusions

In this study, the potential of chemically modified hemp seed hull particles to be used as biofillers in thermoplastic cellulose acetate biocomposites has been investigated. During the alkaline treatment of hemp seed hulls with sodium hydroxide solution, up to 29% of hemicellulose and 9% of lignin were removed from the biomass without the loss of cellulose, demonstrating the strong affinity of sodium hydroxide for amorphous cell wall components and contributing to cellulose enrichment. Thermoplastic biocomposites containing 20 wt% of alkaline-treated hemp seed hull biofillers were achieved through twin-screw extrusion. The most important findings concerning thermoplastic cellulose acetate and hemp seed hull biocomposites could be summarized as follows:

(i) The mechanical testing results confirmed that moderate alkaline treatment of hemp seed hulls using 8–12% NaOH solution yielded the most favourable reinforcement effect in cellulose acetate biocomposites, which demonstrated the best overall performance, combining high tensile strength and stiffness, and superior hardness. The tensile modulus increased by 17–24% (from 1114 MPa to 1306–1377 MPa), and tensile strength improved by 21–23% (from 29.4 MPa to 35.4–36 MPa) in biocomposites with HH8 and HH12 fillers. The hardness of the biocomposites also increased by approximately 11–13%. These findings indicate that controlled alkali modification of the biofiller enhanced fibre–matrix interactions and improved the mechanical robustness of the resulting biocomposites, whereas untreated or overly alkali-treated fillers offered less effective reinforcement;

(ii) The incorporation of particulate alkali-treated hemp seed hulls in the cellulose acetate slightly enhanced the aerobic biodegradation of formed biocomposites due to the removal of hemicellulose, lignin, proteins, and extractives during the treatment, and thereby increased surface accessibility, enabling more effective microbial attack.

These results suggest that alkaline modification of the hemp seed hull biofiller positively affected the mechanical and biodegradation properties of cellulose derivative-based biocomposites. Although this study demonstrates the feasibility of producing cellulose acetate biocomposites with agrowaste filler and highlights their advantages, further research is required to optimize their structure and properties. Future work should include additional thermal processing and mechanical performance studies, long-term durability assessments, and more comprehensive biodegradability testing to evaluate end-of-life scenarios and environmental impacts.

## Figures and Tables

**Table 1:** Thermal processing parameters and mechanical characteristics of CA composites with different hemp seed hull fillers.

Sample	SME (kJ/kg)	MFR (g/10 min)	Tensile Strength (MPa)	Elongation at Break (%)	Tensile Modulus (MPa)	Hardness (Shore D)
CA/TA	542.6 ± 22.6	39.4 ± 0.6	40.75 ± 2.64	6.13 ± 0.09	1153 ± 86	68.4 ± 1.62
CA/TA:HH	168.1 ± 15.5	30.7 ± 0.5	27.88 ± 4.09	2.81 ± 0.21	1110 ± 96	72.4 ± 4.32
CA/TA:HH0	171.20 ± 10.3	30.4 ± 0.5	29.37 ± 4.83	2.81 ± 0.19	1114 ± 42	73.9 ± 2.90
CA/TA:HH8	127.4 ± 14.0	20.3 ± 2.2	35.43 ± 2.50	2.47 ± 0.19	1306 ± 49	74.7 ± 1.72
CA/TA:HH8/Ac	110.1 ± 10.8	16.9 ± 0.3	32.02 ± 1.33	2.19 ± 0.24	1277 ± 38	74.3 ± 2.05
CA/TA:HH/Ac	123.9 ± 12.4	17.4 ± 0.3	31.61 ± 1.87	2.59 ± 0.46	1247 ± 88	73.8 ± 3.41

**Table 2:** Chemical composition of water-washed and alkali-treated hemp seed hull samples.

Sample	Lignin (%)	Cellulose (%)	Hemicellulose (%)	Protein (%)	Fat (%)	Ash (%)
HH0	34.6 ± 0.68	18.7 ± 1.94	24.8 ± 1.24	12.9 ± 0.14	3.5 ± 0.18	2.37 ± 0.04
HH4	32.7 ± 0.26	30.6 ± 1.44	21.5 ±1.08	8.8 ± 0.05	2.9 ± 0.15	2.35 ± 0.02
HH8	32.0 ± 0.03	34.3 ± 1.58	19.2 ± 0.96	7.1 ± 0.33	2.7 ± 0.14	2.15 ± 0.06
HH12	32.6 ± 0.75	36.0 ± 1.28	17.9 ± 0.90	6.4 ± 0.21	2.2 ± 0.11	2.79 ± 0.09
HH16	31.5 ± 1.01	39.5 ± 0.68	17.5 ± 0.88	5.8 ± 0.04	1.7 ± 0.08	1.46 ± 0.06

**Table 3:** Mechanical characteristics of injection moulded CA composites with different hemp seed hull fillers.

Sample	Tensile Strength (MPa)	Elongation at Break (%)	Tensile Modulus (MPa)	Hardness (Shore D)
CA/TA:HH0	29.37 ± 4.83	2.81 ± 0.19	1114 ± 42	65.9 ± 2.90
CA/TA:HH4	27.80 ± 2.91	2.44 ± 0.52	1172 ± 77	70.8 ± 2.08
CA/TA:HH8	35.43 ± 2.50	2.47 ± 0.19	1306 ± 49	74.7 ± 1.72
CA/TA:HH12	36.00 ± 2.55	2.51 ± 0.24	1377 ± 51	73.4 ± 2.15
CA/TA:HH16	34.85 ± 1.87	2.74 ± 0.76	1347 ± 88	69.3 ± 2.41

**Table 4:** The hemp seed hull chemical treatment conditions and sample coding.

Sample Code	Alkaline Treatment			Acetylation Treatment		
	Reagents	T (°C)	Duration (h)	Reagents	T (°C)	Duration (h)
HH0	Distilled water	20 ± 1	24	-	-	-
HH4	4% sodium hydroxide	20 ± 1	24	-	-	-
HH8	8% sodium hydroxide	20 ± 1	24	-	-	-
HH12	12% sodium hydroxide	20 ± 1	24	-	-	-
HH16	16% sodium hydroxide	20 ± 1	24	-	-	-
HH8/Ac	8% sodium hydroxide	20 ± 1	24	Acetic anhydride	30 ± 1	24
HH/Ac	-	-	-	Acetic anhydride	30 ± 1	24

## Data Availability

The original contributions presented in this study are included in the article. Further inquiries can be directed to the corresponding author.
